# Influences on Attitudes Regarding COVID-19 Vaccination in Germany

**DOI:** 10.3390/vaccines10050658

**Published:** 2022-04-22

**Authors:** John Paul Fobiwe, Peter Martus, Brian D. Poole, Jamie L. Jensen, Stefanie Joos

**Affiliations:** 1 Center for Public Health and Health Services Research, University Hospital Tuebingen, 72076 Tuebingen, Germany; stefanie.joos@med.uni-tuebingen.de; 2 Institute for Clinical Epidemiology and Applied Biometry, University Hospital Tuebingen, 72076 Tuebingen, Germany; peter.martus@med.uni-tuebingen.de; 3 Department of Microbiology and Molecular Biology, Brigham Young University, Provo, UT 84602, USA; brian_poole@byu.edu; 4 Department of Biology, Brigham Young University, Provo, UT 84602, USA; jamie.jensen@byu.edu

**Keywords:** COVID-19, vaccine, vaccine acceptance, vaccine hesitancy, vaccine refusal, vaccine attitude

## Abstract

Trust in institutions and democracy may be a major contributor to the willingness to be vaccinated. We investigated these factors and others with regard to COVID-19 vaccine uptake in Germany. Even though effective vaccination is a major contributor to slowing down the current pandemic, vaccine hesitancy remains a major challenge. To analyze attitudes toward vaccine hesitancy, a web-based cross-sectional survey was used to understand and describe the influences of attitudes about vaccination against COVID-19 in the German population. A descriptive analysis for the entire dataset was carried out, and multiple proportional odds regression, path model, and structural equation modeling (SEM) were subsequently used to analyze any relationship between latent variables and COVID-19 vaccine acceptance. In total, 1092 responses from across Germany were analyzed. SEM modeling revealed that trust in institutions, trust in non-pharmaceutical interventions, and various demographic factors were associated with intent to vaccinate. Descriptive analysis and multiple proportional odds regression confirmed that a history of influenza vaccination and level of satisfaction with democratic institutions were highly predictive (*p* < 0.05) for COVID-19 vaccine acceptance. Additionally, social determinants of health such as gender, age, number of children in the family, and the degree of satisfaction with life were also predictors (*p* < 0.05) for COVID-19 vaccine acceptance. Results also demonstrated a significant relationship between receiving the flu vaccine and acceptance of the COVID-19 vaccination. Governments that provide COVID-19 vaccines and control messaging should strive for trust and transparency to maximize vaccine uptake. Government-based vaccine measures should also involve measures to communicate trust in democratic and scientific institutions.

## 1. Introduction

The coronavirus disease 2019 (COVID-19) pandemic has caused severe harm both at individual and public health levels, inflicting many problems in the health status, health systems, and economies of many countries worldwide. Studies prior to COVID-19 vaccine instruction showed that COVID-19 vaccines would make a major contribution to ending this pandemic [1]. However, vaccine hesitancy still remains a major concern since vaccine refusal has been increasing both in Europe and worldwide [2,3]. An effective vaccine campaign contributing to controlling the COVID-19 pandemic through the acquisition of much-needed herd immunity can only be carried out if we understand the reasons and obstacles behind hesitancy for COVID-19 vaccines [4]. The World Health Organization (WHO) even identified vaccine denial as one of the ten greatest threats to global health in 2019, before the COVID-19 pandemic began, and recommended a preventive strategy to improve vaccine adoption and build trust in vaccines to get the maximum effectiveness of vaccinations [5]. Therefore, without understanding these obstacles, we cannot address the potential hesitancy affecting the current COVID-19 vaccination campaign and slowing down the ability to reach herd immunity [6,7,8].

Germany uses all five vaccines approved by the European Union (Biontech Comirnaty, COVID-19 Vaccine Janssen, Nuvaxovid (NVX-CoV2373), Spikevax (COVID-19 Vaccine Moderna), and Vaxzevria (COVID-19 Vaccine AstraZeneca). A full vaccination certificate as per Section 22a of the German Infection Protection Act (IfSG) is proof of the existence of complete vaccination against the coronavirus SARS-CoV-2 if the underlying individual has been vaccinated at least three times with one or more above authorized vaccines and the last single vaccination was given at least three months after the second single vaccination [9].

Data from the COVID-19 Snapshot Monitoring (COSMO) survey in Germany showed that the public is more likely to be vaccinated with the BioNTech vaccine. Moderna also has greater public acceptance than vector-based vaccines such as COVID-19 Vaccine Janssen and Vaxzevria (COVID-19 Vaccine AstraZeneca) [10].

In a recent publication examining obstacles to vaccination in various European countries, it was found that 10% of the German population was strongly against a possible COVID-19 vaccination. According to this study, the willingness to be vaccinated has decreased from 70% to 61% in the past three months. Furthermore, about 20% of the respondents were undecided about a COVID-19 vaccine and would rather not be vaccinated [11]. Similar international studies were carried out before or around the beginning of our study in France [12,13], the USA [14,15,16], Japan [17], Canada [13], and Israel [18] to explore the reasons behind vaccine hesitancy. At the beginning of our study, which was conducted in January 2021, these reasons were not yet exclusively investigated in detail for the German population. One peer-reviewed article exclusively addresses vaccine refusal only amongst health care workers in Germany; this study was also carried out after our survey from 2 February 2021 to 28 February 2021 [19]. An in-depth qualitative and quantitative analysis of the attitudes towards vaccine confidence in the German setting is therefore necessary.

Our hypothesis is that trust in the institutions recommending COVID-19 vaccination has an influence on attitudes towards the potential COVID-19 vaccination. We hypothesize that previous experience with other vaccinations and demographic factors such as age, gender, and income play a role in the decision to be vaccinated. The knowledge from this study is therefore highly relevant for a successful vaccination campaign and for corresponding information and communication policies needed for a successful COVID-19 vaccination program.

## 2. Materials and Methods

### 2.1. Study Sample and Measuring Instruments

We conducted a cross-sectional nationwide anonymous web-based survey of people aged 18 years and above. Respondents were included from each of the 16 federal states. The survey was launched on 4 January 2021 and conducted until 17 January 2021 in the German language. This time period included the peak of the second COVID-19 wave with nationwide incidences of at least 120 cases/100,000 inhabitants. This was shortly after the kickoff of the vaccination campaign, which prioritized vaccination for residents and workers in old people’s and nursing homes, as well as for critical health care workers directly involved in the treatment of COVID-19 patients. At the time the survey was carried out, only the BioNtech/Pfizer and Moderna SARS-CoV-2 vaccines were approved to be used in Germany. The third vaccine, Vaxzevria (AstraZeneca), and the fourth vaccine (Janssen/Johnson/Johnson) were approved after the survey had been completed, Vaxzevria on 11 January 2021 and Johnson/Johnson on 11 March 2021.

The questions asked in our survey instrument were mostly adapted from the standardized protocol of vaccine hesitancy using the 5C model published by Betsch et al. in 2018 [20], a model that has already been proposed by the “WHO Strategic Advisory Working Group of Experts on Immunization (SAGE)” as an instrument for investigating vaccination acceptance or refusal [21].

We hypothesized that previous experience with COVID-19 infection, previous experiences with past vaccines such as the influenza vaccine, and trust in the institutions responsible for promoting vaccination influence attitudes toward COVID-19 vaccination. Vaccine attitude was coded on an ordinal scale with four categories, as shown in Table 1. We also looked at the attitudes towards a COVID-19 vaccination, including vaccine confidence and hesitancy, trusted information sources, experiences with previous vaccination, and personal experience with COVID-19 infection. Covariates such as personal feelings and impact of the COVID-19 pandemic, knowledge about COVID-19, vaccine efficacy, potential side effects, speedy regulatory approval processes, health literacy, trust in science and regulatory authorities, knowledge of vaccine development and technology, trust in government institutions and health care providers, satisfaction with life as well as the impact of the corresponding sociodemographic factors were included in our model so as to provide a more complete explanation for the reasons behind the attitudes about COVID-19 vaccination.

The survey contained 84 items grouped in the following eleven latent variables: experiences with COVID-19 infection; source of information about COVID-19; health literacy about COVID-19 and vaccinations; vaccination history and influenza vaccination; vaccine intentions and attitudes towards COVID-19 vaccines; attitude towards vaccinating of children; impact and influence of the COVID-19 pandemic; world ethics and solidarity with vaccines; knowledge of COVID-19 vaccine development and trust in regulatory agencies; trust in government facilities, health care policies, democracy, and health care institutions; and sociodemographic data. The full questionnaire, including answer options, is found in the supplemental materials (Table S1). The survey was administered by the market research company Qualtrics across Germany after being programmed into the Qualtrics survey tool “ESOMAR”. Qualtrics is well known to have conducted similar surveys in Europe and the United States [22]. A total of 1092 responses were collected. The survey was administered via email notification through the Qualtrics survey panel using an anonymous link, and once the required number of responses was collected, the survey was closed. SEM requires at least 20 subjects per variable. We began the model with 20 variables and covariates, so at least 240 subjects were required. We actually received over 1000 responses. Participants were selected by age in all federal states in Germany. Quality control was performed using a timing method, whereby any participant who spent less than half the meantime completing the survey was rejected. Additional quality control, which identified respondents who had taken part in the survey several times, was carried out with the help of Relevant-ID and did not include the recording of personal data of the respondent. The dataset used was anonymous and prevented the identification of any individual study subject by the research team at any stage of the study. The entire survey is available upon justified request.

### 2.2. Structural Equation Modeling (SEM) and Three-Level Path Modeling

Some of the questions in the survey were adapted for use from Pogue K, Poole B et al. [16], who earlier, in a similar survey in the US population, examined specific latent variables based on a similar hypothesis that these latent variables impact the intention to vaccinate against COVID-19. Since some reports have cited increasing COVID-19 vaccine hesitancy in healthcare workers [13,23,24], we also included healthcare workers in our analysis. The original model design included five latent variables: *attitudes towards vaccination in general, fear of side effects, trust in institutions, trust in non-pharmaceutical interventions, and the effect of COVID-19 on your life*. The model was used to evaluate the influence of these variables on the outcome variable: intent to be vaccinated against COVID-19. Of the 1092 responses to the survey, 1075 completed every question used in the model, so the SEM results are based on these complete responses.

Confirmatory factor analysis revealed that the latent variable *attitudes towards vaccination in general* were associated so completely with intent to be vaccinated that it was essentially impossible to separate the two variables. Similarly, the variable for *fear of side effects* showed too high of collinearity to be separated from the outcome variable. The questions associated with the *effect of COVID-19 on your life* did not show good inter-item consistency and were therefore removed from the analysis. This left two latent variables, *trust in institutions* and *trust in non-pharmaceutical interventions,* to be analyzed, as well as the covariates “health care worker status”, “education”, “age”, “income”, “sex”, “marital status”, and “health.”

Confirmatory factor analysis of this model showed excellent validity. The fit indices (root mean square error of approximation (RMSEA), comparative fit index (CFI), Tucker–Lewis index (TLI), and standardized root mean square residual (SRMR)) were all acceptable (RMSEA, 0.058; CFI, 0.936; TLI, 0.911; SRMR, 0.042). Structural equation modeling was therefore performed utilizing the Mplus software, version 8 (Muthen and Muthen, 1998–2010).

The SEM analysis showed strong associations between several latent variables and intent to vaccinate. Therefore, a 3-level path model was used to refine the understanding of these contributors by determining the extrinsic and mediator variables most closely associated with the intent to vaccinate. 

### 2.3. Statistical Analysis of Individual Variables

All the 1092 participants above 18 years of age who completed the survey were included in the descriptive analysis. Data were evaluated with the statistical program IBM SPSS Statistics Version 27. A descriptive analysis of the distribution of the responses for the entire dataset was carried out. A relationship between certain variables such as vaccination history and prior knowledge of vaccination, basic knowledge of vaccine immunity, prior personal experience of a COVID-19 infection, and pandemic information sources were compared with attitudes and intentions towards a COVID-19 vaccine. Associations were also examined between vaccine intention and standard demographic determinants such as age, gender, level of education, household income, religious affiliation, political attitudes, and work in the health sector. In addition, the effects of trust in relation to the non-pharmaceutical interventions such as masking and social distancing taken by the authorities were also examined. Intent to vaccinate was indicated using an ordinal scale with four variables (Table 1). Chi-square analysis and Pearson’s correlation analysis were used where appropriate. 

## 3. Results

### 3.1. Overall COVID-19 Vaccine Acceptance

In general, 280 (25.6%) of the participants stated that they were willing to be vaccinated against COVID-19 if a vaccine was available to the general public, and another 274 (25.1%) of the participants stated they would be vaccinated after consulting their doctors and being recommended to do so, making a total of 50.7% willing to be vaccinated either through self-autonomy or with the advice of their doctor (Table 1). A total of 346 (31.7%) of the participants said they would “wait to see how the vaccine is working for other people” before being vaccinated themselves, and 192 (17.6%) stated that they did not want to get vaccinated (Table 1). 

### 3.2. Sociodemographic Characteristics and Acceptance of COVID-19 Vaccination

Of the participants, 434 indicated they were male (43.1%), 556 (55.2%) were female, and 17 (1.7%) were not male and not female (Table 2) (*n*-1007). The average age was 33.5 years (range: 18–77 years). As shown in Table 2, we found considerable differences in the willingness to be vaccinated across genders and age groups (*p* < 0.001). A significantly higher proportion of men were willing to get vaccinated or willing to do so if their doctor recommended it (59.2%) as compared to women (44.0%). The highest willingness to be vaccinated was found among the eldest age groups. Regarding marital status, 60.1% (223 out of 338) of those who were married showed a willingness to get vaccinated in contrast to 44.3% (175 out of 386) of people who were not. There was only a weak association to the number of children (*p* = 0.017); however, in the small group with more than three children, only 37% were willing to get vaccinated. There was no difference between the 16 counties (“Bundesländer”) of Germany and between people who claimed to be religious and those who did not (*p* > 0.05). People favoring the conservative (CDU/CSU) and the green party showed the highest willingness to be vaccinated (65.2% and 60.8%), while only 34.4% of those in favor of a right-wing populistic party (AFD) were willing to get vaccinated (*p* < 0.001). Correspondingly, 76% of subjects who were highly satisfied with democracy in Germany (152 out of 200) were willing to get vaccinated, in contrast to only 17.1% (18 out of 105) of subjects who were completely unsatisfied. Education was not associated with willingness, and there was only a weak association with a slight increase in willingness with higher income (*p* = 0.019). 

### 3.3. Subject Characteristics Related to Life Satisfaction and Own Health Status

The respondents’ perception of their own health status was not associated with the willingness to be vaccinated (*p* > 0.05). However, 66.4% (166 out of 250) of subjects affected by risk factors for COVID-19 were willing to be vaccinated compared to 46.2% (323 out of 700) not affected by risk factors (*p* < 0.001). Importantly, 8.4% of people classified as high-risk patients for COVID-19 infection say they would not or probably would not get vaccinated, as do 13.5 % of those who say they are health care workers (Table 3). There was a highly significant association with life satisfaction: 63.1% of subjects who were highly satisfied with life were willing to be vaccinated vs. 21.3% of subjects who were not totally satisfied (*p* = 0.0002) (Table 4).

### 3.4. Subject Characteristics Related to Vaccination and COVID-19

Receiving a positive test for COVID-19 was a strong predictor in favor of vaccination (68.6% vs. 49.6%, *p* = 0.006). Additionally, the effect of the pandemic on the daily life of participants was not related to the willingness to get vaccinated. Acceptance of non-pharmaceutical interventions against the pandemic (masks, distance, hand washing, use of warning apps, reduction of social contacts) was highly associated with willingness for vaccination. Regarding health-related vs. economic aspects, those who gave priority to activities against the virus were much more willing to be vaccinated (66.7%) compared to those who gave priority to the economy (24.3%) (*p* < 0.001).

As expected, characteristics referring to vaccination and COVID-19, in general, were highly associated with willingness to be vaccinated. These factors included attitude towards vaccination in general and especially flu vaccination, satisfaction with information about the pandemic, one’s own knowledge about the pandemic, opinion of challenges of the pandemic, trust in information from official sources and medical doctors, and positive attitude towards support for developing countries regarding vaccination. Those who received information primarily from social media and other sources tended to be more vaccine-hesitant.

### 3.5. Statistical Modeling 

#### 3.5.1. SEM

Given the large number of associations between individual survey questions and vaccination willingness, SEM was used to test a model determining how variables associated with these questions correlated with willingness to be vaccinated. This model was used by Pogue et al. [16] and was used here to test whether those findings also applied in Germany. The variable *attitude towards vaccination in general* correlated almost perfectly with *intent to get the COVID-19 vaccine*. To examine other variables, the *attitude towards vaccination in general* variable was removed, and the model was run without these questions. 

**Figure 1:**
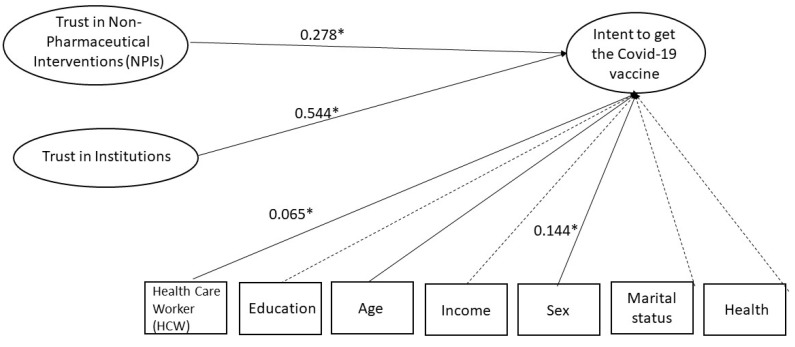
SEM evaluating factors involved in intent to receive the COVID-19 vaccine. Trust in Non-pharmaceutical interventions such as masks and social distancing was significantly associated with intent to receive the COVID-19 vaccine, as was trust in institutions such as German health organizations and democracy in Germany. Several covariates also showed significant association. Being a health care worker, older age, and male sex were all associated with increased intent to be vaccinated. Attitude toward all vaccines, including vaccine history, was so strongly associated with intent to get the COVID-19 vaccine that it masked other interactions, and so it was removed from the model. Solid lines indicate significant associations, while dashed lines did not reach significance. Numbers next to the variable names show the survey questions associated with each variable. * Indicates significance at < 0.05.

The model found a strong association between *trust in institutions* (medical institutions and governmental institutions were grouped together for this variable) and *intent to get the COVID-19 vaccine* (Figure 1). There was also a strong association between *trust in non-pharmaceutical interventions* (for example, masks and distancing) and willingness to be vaccinated. Several demographic covariates were included in the model. Of these, increased age and male sex were associated with increased willingness to be vaccinated. Being a healthcare worker was slightly associated with increased willingness. No significant associations were found for education, income, marital status, or health.

#### 3.5.2. Multivariate Analysis and Path Model

**Figure 2:**
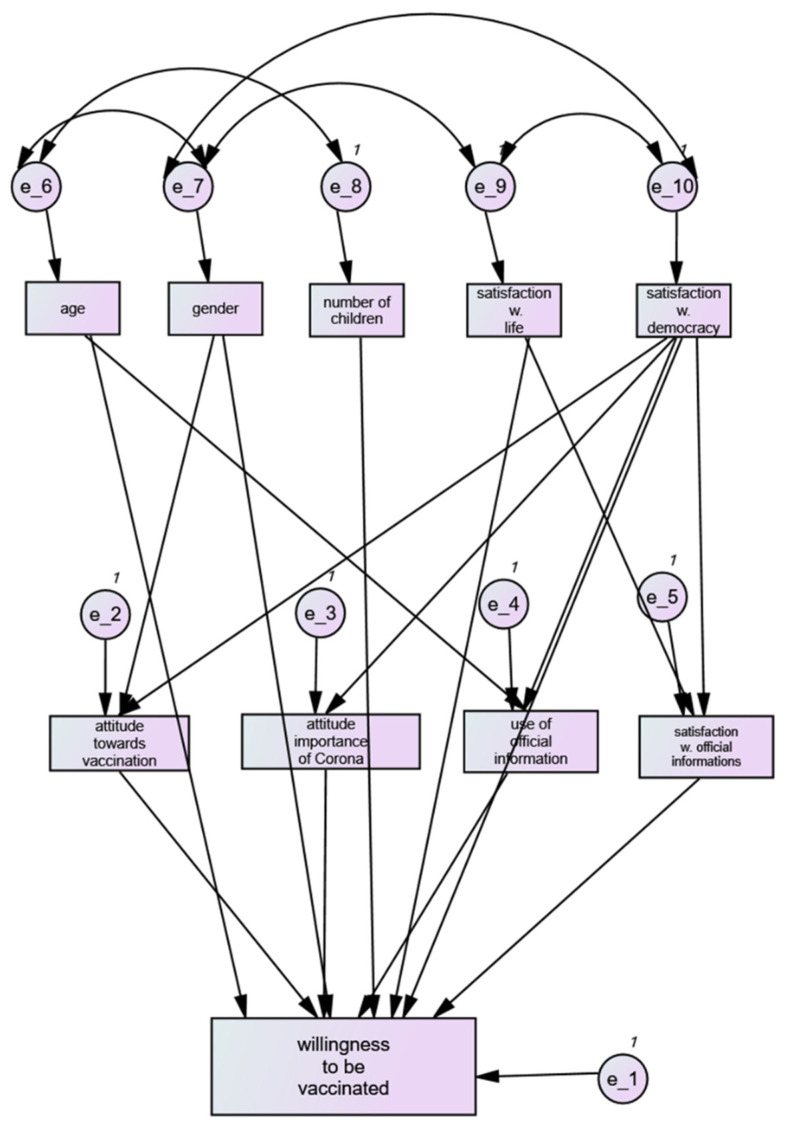
Three-level path model. The extrinsic variables were those questions not directly related to COVID-19 (top row), and the mediator variables were those directly related to COVID-19 attitudes (second row). Variables with the strongest association with willingness to be vaccinated are satisfaction with democracy (r = 0.48), attitude towards vaccination (r = 0.40, satisfaction with official information (r = 0.38), and attitude towards the importance of COVID-19 (r = 0.27). e_1 to e_10 are error terms for residual variances needed to complete the model, as a formal part of the model.

Two proportional odds models for the vaccine attitude were fitted. In the first model, only factors that are not directly related to vaccination but characterize the general characteristics of participants were included. Significant predictors of less willingness to be vaccinated were younger age, female gender, number of children, satisfaction with life, and satisfaction with democracy (Table 4). In the second model, less importance of the COVID-19 disease, less vaccination against influenza, less satisfaction with information regarding COVID-19, and less use of official sources of information were significant (Table 5). The approach of differentiating between general and COVID-19-related factors motivated the construction of the path model by using the latter factors as mediators instead of extrinsic factors (Figure 2).

### 3.6. Results of the Path Model

In a three-level path model, predictors independent from COVID-19 were used as extrinsic variables, predictors related to COVID-19 as mediators, and willingness to be vaccinated was used as outcome. The strongest extrinsic variable was satisfaction with democracy (r = 0.42) followed by gender (0.20), satisfaction with life (0.20), age (0.16), and number of children (0.08). The most relevant mediator variables were the attitude toward vaccination (0.40) and satisfaction with official information about COVID-19 (0.38), followed by the attitude towards the importance of COVID-19 (0.27) and the use of official information (0.07).

## 4. Discussion

We found that trust in democracy and institutions significantly predicted the respondents’ attitudes towards a potential COVID-19 vaccine, results which were confirmed with every model we used. Additionally, social determinants such as gender, age, number of children in the family, and the degree of satisfaction with life were also predictors for a COVID-19 vaccine. Trust in vaccines in general, including actions such as previously receiving the flu vaccine, was strongly associated with COVID-19 vaccination, as shown by both analysis of individual questions, path modeling, and the SEM model. The results of each analysis are similar in that they all point to trust in society, science, and societal institutions as being determinative of intent to receive the COVID-19 vaccine. Since COVID-19 skeptics have long been critical of the anti-pandemic measures [25] and with new evidence both worldwide and within the EU (Public Opinion Monitoring Unit) now indicating a negative shift with regard to perceptions of democracy alongside public health trust [26,27], one of the most important ingredients for an effective vaccine campaign is to build up both trust in the vaccine authorities and trust in the democratic principles of government. This is particularly relevant since vaccine hesitancy did not start with COVID-19 vaccine hesitancy.

These results may help to explain the current COVID-19 snapshot monitoring study in Germany, which shows that as of July 2021, 10% are strictly against the COVID-19 vaccination under any circumstances. Among the unvaccinated in the COVID-19 snapshot monitoring, vaccine hesitancy is mainly due to safety reasons (41% of unvaccinated) [28]. Additionally, the ongoing COVIMO study [29] of the Robert Koch Institute (RKI) aimed to continuously capture vaccination behavior, willingness, and acceptance of COVID-19 vaccines in Germany, as well as other international capture studies such as the US COVID-19 Vaccine Monitor study of the KFF (Kaiser Family Foundation) [30], share similar observations that people who are more hesitant about the COVID-19 vaccine show a decreasing willingness to get vaccinated over time, again pointing to the need to analyze the reasons behind this hesitancy as our study did. 

It is, therefore, extremely important to ensure transparency in the information provided about the COVID-19 vaccines in terms of not only their efficacy but also side effects and information about the institutions that review and approve these vaccines to maintain maximum trust. Government campaigns to increase vaccination should be focused on making it clear that the vaccines reviewed by government and scientific institutions are safe and that these vaccines also protect others, therefore making it possible to achieve the urgently desired herd immunity of above 80 percent (85% for the age groups 12–59 years and 90% for ≥60 years) as calculated by Robert-Koch-Institute for Germany [31].

With regards to VOCs, public opinion polling shows that the majority of people are concerned about post-vaccination protection and vaccine breakthroughs. In fact, the December 2021 survey showed that the effectiveness achieved is an important factor in vaccination readiness. The majority of the public thinks that vaccine effectiveness is negatively affected by VOCs, and the majority of those who share concerns about VOCs show a greater willingness to get a booster shot [10,32].

Since there was some indication that large families are more reluctant to vaccinate, we propose that a clear vaccination campaign about the safety and effectiveness issues of the vaccine while building up trust in government institutions may be needed, especially when it comes to protecting our children. Vaccine coverage in children is still low, and for them, it is very relevant that adults are vaccinated.

### Limitations

Our study has some limitations. Firstly, selection bias cannot be eliminated since this study was a web-based survey. Secondly, this study was a cross-sectional study carried out in the middle of the second wave (January 2021); therefore, we cannot rule out decision changes due to the second wave, and consequently, no causality could be established. 

Despite these limitations, our findings are quite unique and novel in using path modeling and SEM to measure the attitudes towards COVID-19 vaccination in Germany. We found that not just trust in the vaccine but also efforts to increase trust in our basic public policies and democratic values would be important to target vaccine hesitancy. Ending this COVID-19 pandemic through vaccination will require thorough communication to dispel disinformation and fundamental misunderstandings about COVID-19 and vaccine safety alongside clear communication of reasons to trust in democracy. As more people are protected from COVID-19, vaccines will allow us to return to normal physical interactions as travel restrictions, masking, and distancing requirements are lifted, as vaccines have for decades. The strong extrinsic association of trust in democracy indicates that future studies should address the association between democracy and vaccine uptake.

## 5. Conclusions

The main factor that stood out in this analysis was the correlation between trust in Democracy and willingness to be vaccinated. There was a highly significant association between these variables in individual correlations, in the path model, and in the SEM. Trust in democratic and state institutions is therefore extremely important when asking people to be vaccinated and trust in public health information, such as the usefulness of masks and social distancing. Efforts should be put into increasing trust in institutions to improve vaccine attitudes. 

## Figures and Tables

**Table 1:** COVID-19 vaccine attitudes.

When a Vaccine for COVID-19 Is Approved and Widely Available to Anyone Who Wants It, Respondent Will: (*n* = 1092)	Percentage of Sample
Get the vaccine immediately	25.6
Only get the vaccine after consulting with doctor	25.1
Wait until it has been available for a while to see how it is working for other people	31.7
Definitely not get the vaccine	17.6

**Table 2:** Demographics and vaccine attitudes. Where *n* < 1092, some participants did not answer the question.

Total Respondents N = 1092	N	%	Will Be Vaccinated Right Away (%)	Will Be Vaccinated after Consulting Doctor (%)	Will Wait and See How Others Tolerate Vaccination (%)	Will Not Be Vaccinated at All (%)	
Gender (*n* = 1007)							
Male	434	43.1	35.0	26.5	26.3	12.1	*p* (<0.001)
female	556	55.2	20.5	25.5	34.4	19.6	
Not male/Not female	17	1.7	5.9	35.3	23.5	35.3	
No. children in the family (*n* = 1092)							
Non	612	56.0	28.4	23.5	32.7	15.4	*p* (<0.001)
One child	232	21.2	15.9	13.2	18.3	8.6	
Two children	172	15.8	23.3	29.7	34.9	12.2	
Three children	49	4.5	24.5	24.5	24.5	26.5	
More than three children	27	2.5	7.4	29.6	22.2	40.7	
Age group (years) (*n* = 1092)							
18–25	435	39.8	18.6	28.5	33.6	19.3	*p* (<0.001)
26–35	273	25.0	23.8	23.8	33.3	19.0	
36–45	157	14.4	29.3	21.0	27.4	22.3	
46–55	122	11.2	34.4	23.0	33.6	9.0	
56–65	66	6.0	40.9	25.8	27.3	6.1	
66-77	39	3.6	48.7	17.9	17.9	15.4	
Household income (*n* = 1078)							
EUR < 1250	229	21.2	25.8	22.7	31.9	19.7	*p* (<0.058)
EUR 1250–1750	168	15.6	19.0	31.5	33.9	15.5	
EUR 1750–2250	182	16.9	19.8	28.0	30.2	22.0	
EUR 2250–3000	197	18.3	25.9	25.9	31.0	17.3	
EUR 3000–4000	168	15.6	39.8	25.0	33.9	11.3	
EUR 4000–5000	62	5.8	32.3	16.1	30.6	21.0	
EUR ≥ 5000	72	6.7	40.3	15.3	29.2	15.3	
Education (*n* = 1083)							
Never completed school	21	1.9	23.8	28.6	28.6	19.0	*p* (<0.001)
Elementary school	173	16.0	28.9	22.0	29.5	19.7	
Secondary school	352	32.5	23.0	26.1	31.8	19.0	
Technical school diploma	112	10.3	22.3	24.1	38.4	15.2	
High-school diploma	198	18.3	25.3	27.8	31.8	15.2	
Some college or university of applied sciences degree	83	7.7	28.9	22.9	32.5	15.7	
Completed college or university of applied sciences degree	122	11.3	31.1	26.2	29.5	13.1	
Doctoral degree	22	2.0	22.7	13.6	27.3	36.4	
Marital status *n* = 1088							
Not married	386	35.5	22.0	23.3	35.2	19.4	*p* (<0.001)
Married	338	31.1	36.1	24.0	27.2	12.7	
Living with a partner in a steady relationship	269	24.7	19.0	27.5	34.6	19.0	
Widowed	28	2.6	14.3	42.9	25.0	17.9	
Divorced	67	6.2	25.4	23.9	23.9	26.9	

The bold is an indicator of different questions.

**Table 3:** Health characteristics and COVID-19 vaccine attitudes.

	N	%	Will Be Vaccinated Right Away (%)	Will Be Vaccinated after Consulting Doctor (%)	Will Wait and See How Others Tolerate the Vaccine (%)	Will Not Be Vaccinated at All	
**Higher risk perception of severe COVID-19 (*n* = 1073)**							
Yes	250	23.3	36.0	30.4	25.2	8.4	*p*(<0.052)
No	700	65.2	23.6	22.6	34.3	19.6	
Not sure	123	11.5	17.1	29.3	30.1	23.6	
**Healthcare workers (*n* = 1068)**							
Yes	222	20.8	22.1	31.5	32.9	13.5	*p* (<0.040)
No	846	79.2	26.8	23.6	31.3	18.2	
**Well-being health status (*n* = 1068)**							
Very good	345	31.9	29.9	22.3	28.1	19.7	*p* (<0.002)
Good	450	41.6	24.7	26.0	34.7	14.7	
Fair	184	17.0	22.3	26.1	38.6	13.0	
Poor	63	5.8	17.5	31.7	23.8	27.0	
Very poor	41	3.8	29.3	24.4	12.2	34.1	

The bold is to identify categories.

**Table 4:** Personal characteristics not directly related to COVID-19 and attitude towards vaccination.

Factor	Odds Ratio ^1^	95% CI	*p*-Value
Age (years)	0.98	0.97–0.99	<0.0001
Female gender vs. male gender	1.65	1.30–2.08	<0.0001
Neither female nor male gender vs. male gender	1.59	0.68–3.74	0.29
Number of children	1.14	1.02–1.28	0.017
Satisfaction with life (four-point Likert scale) ^2^	1.35	1.15–1.59	0.0002
Satisfaction with democracy (four-point Likert scale) ^2^	2.63	2.28–3.04	<0.0001 ^3^

^1^ Odds ratios obtained from a proportional odds model; values > 1 indicate an increased probability not to be vaccinated; ^2^ higher values indicate less satisfaction; ^3^ this *p*-value was <10^−37^.

**Table 5:** Personal characteristics related to COVID-19 and attitude towards vaccination.

Factor	Odds Ratio ^1^	95% CI	*p*-Value
Less importance of COVID-19	1.40	1.29–1.51	<0.0001
No vaccination against influenza	1.99	1.80–2.19	<0.0001
Satisfaction with information ^2^	1.44	1.28–1.62	<0.0001

Odds ratios from a proportional odds model; ^1^ values > 1 indicate an increased probability not to be vaccinated; ^2^ higher values indicate less satisfaction.

## Data Availability

Data can be obtained by valid request to the corresponding author.

## Supplementary Materials

The following are available online at https://www.mdpi.com/article/10.3390/vaccines10050658/s1, Table S1: Survey questions.
