# Supplementary material for: Influences on Attitudes Regarding COVID-19 Vaccination in Germany
Source: Vaccines (Basel). 2022 Apr 22;10(5):658. doi: 10.3390/vaccines10050658 (PMC9146249; doi:10.3390/vaccines10050658)
Supplement: Supplementary file 1 [file vaccines-10-00658-s001.zip › vaccines-1662171-supplementary.pdf]

Block 1: Erfahrungen mit COVID-19-Infektion (7 Fragen)

1. **Bitte wählen Sie durch Ankreuzen die Aussagen aus, die auf Ihre Erfahrungen mit COVID-19 zutreffen (Mehrfachauswahl möglich):**
    - Ich wurde positiv auf eine COVID-19-Infektion getestet. (1)
    - Ein nahes Familienmitglied oder eine Person im gleichen Haushalt wurde positiv auf COVID-19-Infektion getestet (2)
    - Ein entferntes Familienmitglied wurde positiv auf COVID-19 getestet (3)
    - Ein Bekannter, der nicht im gleichen Haushalt wohnt, wurde positiv auf COVID-19 getestet (4)
    - Ein Mitarbeiter oder Arbeitskollege wurde positiv auf COVID-19 getestet (5)
    - Ein entfernter Bekannter bzw. ein Bekannter eines Bekannten wurde positiv auf eine COVID-19-Infektion getestet (6).
    - Ich kenne niemanden, der positiv auf COVID-19 getestet wurde (7)

Diese Frage anzeigen Falls 1= Ich wurde positiv auf COVID-19-Infektion getestet.

2. **Falls Sie selber positiv getestet wurde, wie schwer war Ihre COVID-19-Infektion?**
    - Keine Symptome / milde Symptome (1)
    - Mäßige Symptome, aber ärztliche Beratung wurde nicht in Anspruch genommen (2)
    - Mäßige Symptome und ärztliche Beratung wurde in Anspruch genommen (3)
    - Schwere Symptome mit Krankenhausaufenthalt Intensivstation(4)

Diese Frage anzeigen Falls 1= ein nahes Familienmitglied oder eine Person im gleichen Haushalt wurde positiv auf COVID-19 getestet (Falls es mehr als einen positiven Fall gab, beschreiben Sie bitte die Symptome der Person, die eine schwerere Krankheit hatte)

3. **Wie schwerwiegend waren die Symptome der COVID-19-Infektion Ihres nahen Familienmitglieds oder bei der Person im gleichen Haushalt?**
    - Keine Symptome / milde Symptome (1)
    - Mäßige Symptome, aber ärztliche Leistung wurde nicht in Anspruch genommen (2)
    - Mäßige Symptome und ärztliche Leistung wurde in Anspruch genommen (3)
    - Schwere Symptome / Krankenhausaufenthalt (4)
    - Verstorben (5)

Diese Frage anzeigen Falls Frage 1 = Ein entferntes Familienmitglied wurde positiv auf COVID-19-Infektion getestet

4. **Wie schwerwiegend waren die Symptome der COVID-19-Infektion Ihres entfernten Familienmitglieds?**
    - Keine Symptome / milde Symptome (1)
    - Mäßige Symptome, aber ärztliche Leistung wurde nicht in Anspruch genommen (2)
    - Mäßige Symptome und ärztliche Leistung wurde in Anspruch genommen (3)
    - Schwere Symptome / Krankenhausaufenthalt (4)
    - Verstorben (5)

Diese Frage anzeigen Falls 1= Ein Bekannter, der nicht im gleichen Haushalt wohnt, wurde positiv auf COVID-19-Infektion getestet

5. **Wie schwerwiegend waren die Symptome der COVID-19-Infektion Ihres Bekannten, der nicht im gleichen Haushalt wohnt?**
    - Keine Symptome / milde Symptome (1)
    - Mäßige Symptome, aber ärztliche Leistung wurde nicht in Anspruch genommen (2)
    - Mäßige Symptome und ärztliche Leistung wurde in Anspruch genommen (3)
    - Schwere Symptome / Krankenhausaufenthalt (4)
    - Vestorben (5)

Diese Frage anzeigen Falls 1= Ein Mitarbeiter oder Arbeitskollege, wurde positiv auf COVID-19-Infektion getestet

6. **Wie schwerwiegend waren die Symptome der COVID-19-Infektion Ihres Mitarbeiters oder Arbeitskollegen?**
    - Keine Symptome / milde Symptome (1)
    - Mäßige Symptome, aber ärztliche Leistung wurde nicht in Anspruch genommen (2)
    - Mäßige Symptome und ärztliche Leistung wurde in Anspruch genommen (3)
    - Schwere Symptome / Krankenhausaufenthalt (4)
    - Verstorben (5)

Diese Frage anzeigen Falls 1= Ein entfernter Bekannter bzw. ein Bekannter eines Bekannten wurde positiv auf eine COVID-19-Infektion getestet.

7. **Wie schwerwiegend waren die Symptome der COVID-19-Infektion Ihres entfernten Bekannten bzw. des Bekannten Ihres Bekannten?**
    - Keine Symptome / milde Symptome (1)
    - Mäßige Symptome, aber ärztliche Leistung wurde nicht in Anspruch genommen (2)
    - Mäßige Symptome und ärztliche Leistung wurde in Anspruch genommen (3)
    - Schwere Symptome / Krankenhausaufenthalt (4)
    - Verstorben (5)

Block 2: Informationsquelle über COVID-19 (2 Fragen).

1. Woher beziehen Sie die meisten Informationen zu COVID-19? (Bitte geben Sie max. 3 Antworten an)

- Gesundheitsbehörden (Gesundheitsamt/RKI (Robert-Koch-Institut), WHO (Weltgesundheitsorganisation)(1)
- Hausarzt (2)
- Familie, Freunde und Bekannte (3)
- Öffentlich-rechtliche Nachrichtensendungen (4)
- Regionale und lokale Nachrichten (Tageszeitung, Radio oder Regional- und Lokalfernsehprogramme (3)
- Online Nachrichtenportale
- soziale Medien (4)
- Prominente / Persönlichkeiten des öffentlichen Lebens (z.B. Talkshows) (5)
- Religionsverantwortlicher (6)
- Politiker(7)
- Sonstiges (bitte angeben) (8)---

2. Was drückt Ihre Haltung zu den Informationen zur Coronapandemie am ehesten aus?

- Ich finde die Informationsvielfalt gerade richtig.
- Ich wünsche mir noch mehr Informationen.
- Ich fühle mich durch die vielen Informationen eher überfordert.
- Ich fühle mich durch die vielen Informationen eher gelangweilt

Block 3: Gesundheitskompetenzen über COVID-19 und Impfungen (5-Fragen).

1. Wie oft brauchen Sie Hilfe, wenn Sie Anweisungen, Broschüren oder anderes schriftliches Material von Ihrem Arzt oder von Ihrer Apotheke lesen?

- nie
- selten
- gelegentlich
- oft
- Immer

Bitte kreuzen Sie an, ob die folgenden Aussagen richtig oder falsch sind

2. Bestimmte Antibiotika können COVID-19 verhindern und/oder behandeln.

- Richtig (1)
- Falsch (2)
- weiß ich nicht

3. **Im Durchschnitt dauert es 5 - 6 Tage ab einer Infektion mit COVID-19, bis Symptome auftreten. Es kann jedoch auch bis zu 14 Tage dauern.**
   - Richtig (1)
   - Falsch (2)
   - weiß ich nicht

4. **Bei einigen Menschen kann es zu schweren Verläufen der COVID-19-Erkrankung kommen.**
   - Richtig (1)
   - Falsch (2)
   - weiß ich nicht

Bitte geben Sie bei folgenden Aussagen an, wie sehr sie ihnen zustimmen bzw. nicht zustimmen

5. **Impfstoffe sind wichtig für die Prävention schwerer Krankheiten:**
   - Stimme voll und ganz zu (1)
   - Zustimmen (2)
   - Weder noch (3)
   - Nicht einverstanden (4)
   - stimme überhaupt nicht zu (5)

6. **Die gleichzeitige Verabreichung von mehr als einem Impfstoff ist sicher.**
   - Stimme voll und ganz zu (1)
   - stimme zu (2)
   - weder noch (3)
   - Stimme nicht zu (4)
   - Stimme überhaupt nicht zu (5)

7. **Eine COVID-19-Impfung kann sehr hilfreich für die Beendigung der Pandemie sein.**
   - Stimme voll und ganz zu (1)
   - stimme etwas zu (2)
   - Weder noch (3)
   - stimme etwas nicht zu (4)
   - stimme überhaupt nicht zu (5)

8. **Impfungen tragen dazu bei, eine Gemeinschaft bzw. eine ganze Bevölkerung vor Krankheiten zu schützen, vor allem wenn die überwiegende Mehrheit der Bevölkerung geimpft ist (Gemeinschaftsschutz).**
   - stimme voll und ganz zu (1)
   - stimme etwas zu (2)
   - Weder noch (3)

- stimme etwas nicht zu (4)
- stimme überhaupt nicht zu (5)

Block 4: Impfanamnese und Influenza-Impfung (6 Fragen)

1. **Wie hat die aktuelle COVID-19-Pandemie Ihre Meinung zum Impfen im Allgemein verändert?**
   - Es ist viel wahrscheinlicher, dass ich mich impfen lasse (1)
   - Es ist etwas wahrscheinlicher, dass ich mich impfen lasse (2)
   - Meine Meinung zu Impfungen hat sich nicht geändert (3)
   - Es ist etwas weniger wahrscheinlich, dass ich mich impfen lasse (4)
   - Es ist viel weniger wahrscheinlich, dass ich mich impfen lasse (5)
2. **Wie wichtig ist es für Sie, sich jedes Jahr gegen Grippe impfen zu lassen?**
   - Sehr wichtig (1)
   - Wichtig (2)
   - Weder wichtig noch unwichtig (3)
   - Nicht wichtig (4)
   - Überhaupt nicht wichtig (5)
3. **Hat die Covid-19-Pandemie einen Einfluss, ob Sie sich gegen Grippe (Influenza) impfen lassen?**
   - Ja, es ist wahrscheinlicher, dass ich mich gegen Grippe impfen lasse
   - Nein, die Covid-19-Pandemie hat darauf keinen Einfluss
   - Ja, es ist weniger wahrscheinlich geworden, dass ich mich gegen Grippe impfen lasse.
4. **Werden Sie sich in dieser Saison gegen Grippe impfe lassen bzw. haben Sie sich in dieser Saison bereits impfen lassen?**
   - ja
   - nein
   - weiß nicht
5. **Falls Antwort "Ja" ist, Was ist der Hauptgrund, dass Sie sich gegen Grippe impfen lassen? (Bitte nur eine Antwort ankreuzen.)**
   - Ich möchte mich selbst vor einer Influenzainfektion schützen
   - Ich möchte Familienmitglieder, Kollegen oder Klienten/Kunden/Patienten vor einer Infektion schützen
   - Einfacher Zugang zum Impfstoff / Mein Arbeitgeber bietet kostenlose Impfungen gegen saisonale Grippe an
   - Ich gehöre zur Risikogruppe

6. Falls Antwort „Nein“ ist? Was ist der Hauptgrund, dass Sie sich nicht gegen Grippe impfen lassen würden (Bitte nur eine Antwort auswählen)

- Ich gehöre nicht zur Influenza-Infektionsrisikogruppe
- Ich habe Zweifel an der Wirksamkeit des Impfstoffs
- Ich befürchte Nebenwirkungen des Impfstoffs
- Zeitmangel
- Ich habe mich bisher nicht mit der Impfung und ihren Vor- und Nachteilen beschäftigt.
- Schlechte Verfügbarkeit von Impfstoffen

Block 5: Inanspruchnahme der COVID-19-Impfung (9 Fragen).

Bitte geben Sie an, wie Sie in den folgenden Szenarien reagieren würden, wenn ein Impfstoff entwickelt würde, um eine Ansteckung mit COVID-19 zu verhindern.

1. Wenn ein COVID-19-Impfstoff für alle verfügbar wäre...

- Werde ich mich sofort impfen lassen (1)
- Werde ich mich nach Rücksprache und Empfehlung meines Arztes impfen lassen (2)
- Werde ich erstmal abwarten und beobachten, wie viele andere ihn vertragen, damit ich weiß, dass er sicher ist (3)
- Werde ich mich nicht impfen lassen (4)

Falls Frage 1 nicht gleich: Ich wer ich mich sofort impfen lassen

2. Was ist der Hauptgrund, dass Sie sich gar nicht oder nicht sofort impfen lassen? Bitte nur 1 Antwort ankreuzen

- Ich glaube nicht, dass der Impfstoff sicher ist (1)
- Ich glaube nicht, dass der Impfstoff wirksam ist (2)
- Ich vertraue den behördlichen Empfehlungen für die Impfung nicht (3)
- Ich glaube grundsätzlich nicht an den Nutzen von Impfungen, und das gilt auch für einen neuen COVID-19-Impfstoff. (4).
- Ich habe vertrauenswürdige Empfehlungen, mich nicht zu impfen (5)
- Die Impfung ist mir nicht wichtig. (6).
- Anderer Grund (7)-----------------------------

3. **Falls ein COVID-19-Impfstoff öffentlich zugänglich gemacht würde, aber jährlich verabreicht werden müsste (ähnlich wie bei der Grippeimpfung), wie wahrscheinlich wäre es, dass Sie sich impfen lassen?**
    - Sehr wahrscheinlich (1)
    - Wahrscheinlich (2)
    - Etwas wahrscheinlich (3)
    - Nicht sehr wahrscheinlich (4)
    - Überhaupt nicht wahrscheinlich (fast nie) (5)
4. **Falls ein Impfstoff gegen COVID-19 zur Verfügung gestellt würde und Ihnen mitgeteilt würde, dass er die Hälfte (50%) der Personen schützen würde, die ihn erhalten haben, wie wahrscheinlich wäre es, dass Sie geimpft werden?**
    - Sehr wahrscheinlich (1)
    - Etwas wahrscheinlich (2)
    - Weder wahrscheinlich noch unwahrscheinlich (3)
    - Etwas unwahrscheinlich (4)
    - Sehr unwahrscheinlich (5)
5. **Falls ein Impfstoff gegen COVID-19 zur Verfügung gestellt würde und Ihnen mitgeteilt würde, dass er 3/4 (75%) der Empfänger schützen würde, wie wahrscheinlich wäre es, dass Sie geimpft werden?**
    - Sehr wahrscheinlich (1)
    - Etwas wahrscheinlich (2)
    - Weder wahrscheinlich noch unwahrscheinlich (3)
    - Etwas unwahrscheinlich (4)
    - Sehr unwahrscheinlich (5)
6. **Falls ein Impfstoff gegen COVID-19 zur Verfügung gestellt würde und Ihnen mitgeteilt würde, dass er 99% derjenigen schützen würde, die ihn erhalten haben, wie wahrscheinlich wäre es, dass Sie geimpft werden?**
    - Sehr wahrscheinlich (1)
    - Etwas wahrscheinlich (2)
    - Weder wahrscheinlich noch unwahrscheinlich (3)
    - Etwas unwahrscheinlich (4)
    - Sehr unwahrscheinlich (5)

BLOCK 6: Einstellung zur Impfung von Kindern:

Als Filterfrage gedacht: Haben sie Kinder? Wenn Ja wie beurteilen Sie folgende Aussagen zur Impfung von Kindern.

1. **Inwiefern stimmen Sie folgende Aussage zu: Meine Kinder haben alle vom Hausarzt/Kinderarzt empfohlen Impfstoffe erhalten.**
   - stimme voll und ganz zu (1)
   - stimme etwas zu (2)
   - Teils/teils (3)
   - stimme etwas nicht zu (4)
   - stimme überhaupt nicht zu (5)

2. **Würden Sie Ihre Kinder gegen COVID-19 impfen lassen?**
   - Ich würde sie impfen lassen (1)
   - Ich würde sie nicht impfen lassen (2)
   - Ich weiß es nicht. (3)

Diese Frage Anzeigen Falls Frage 2 = Ich habe Kinder und würde sie nicht impfen

3. **Was ist der Hauptgrund, dass Sie Ihre Kinder nicht impfen lassen würden:**
   - Ich glaube nicht, dass der Impfstoff für mein/e Kind/er sicher ist (1)
   - Ich glaube nicht, dass der Impfstoff für mein/e Kind/er wirksam ist (2)
   - Ich vertraue den behördlichen Empfehlungen für die Impfung für mein/e Kind/er nicht (3)
   - Ich glaube grundsätzlich nicht an den Nutzen von Impfungen für mein/e Kind/er, und das gilt auch für einen neuen COVID-19-Impfstoff. (4).
   - Ich habe vertrauenswürdige Empfehlungen, mein/e Kind/er nicht zu impfen (5)
   - Die Impfung für mein/e Kind/er ist mir nicht wichtig. (6).
   - Anderer Grund (7)--

7. **Wie hat die aktuelle COVID-19-Pandemie Ihre Meinung zum Impfen verändert?**
   - Es ist viel wahrscheinlicher, dass ich meine Kinder impfen lasse (1)
   - Es ist etwas wahrscheinlicher, dass ich meine Kinder impfen lasse (2)
   - Meine Meinung zu Impfungen bei Kindern hat sich nicht geändert (3)
   - Es ist etwas weniger wahrscheinlich, dass ich meine Kinder impfen lasse (4)
   - Es ist viel weniger wahrscheinlich, dass ich meine Kinder impfen lasse (5)

8. **Wie wichtig ist es für Sie, Ihre Kinder jedes Jahr gegen Grippe impfen zu lassen?**
    - Sehr wichtig (1)
    - Wichtig (2)
    - Weder wichtig noch unwichtig (3)
    - Nicht wichtig (4)
    - Überhaupt nicht wichtig (5)

9. **Hat die Covid-19-Pandemie einen Einfluss, ob Sie Ihre Kinder gegen Grippe (Influenza) impfen lassen?**
    - Ja, es ist wahrscheinlicher, dass ich meine Kinder gegen Grippe impfen lasse
    - Nein, die Covid-19-Pandemie hat darauf keinen Einfluss
    - Ja, es ist weniger wahrscheinlich geworden, dass ich meine Kinder gegen Grippe impfen lasse.

Block 7: Einfluss der COVID-19-Pandemie (2 Fragen).

1. **Wie hat sich die COVID-19-Pandemie auf Ihr Einkommen ausgewirkt?**
    - Deutlich verschlechtert
    - Etwas verschlechtert(2)
    - Überhaupt keine Änderung (3)
    - Etwas verbessert (3)
    - Deutlich verbessert (5)
2. **Wie sehr stimmen Sie der folgenden Aussage zu bzw. nicht zu? Ich fühle mich in meinen normalen täglichen Aktivitäten durch die Corona-Pandemie stark eingeschränkt.**
    - Stimme stark zu
    - stimme zu
    - weder noch
    - Stimme nicht zu
    - Stimme überhaupt nicht zu

Block 8: Weltethik und Solidarität (2 Fragen).

1. **Reiche Länder sollten dafür sorgen, dass ein COVID-Impfstoff kostenlos für ärmere Länder zur Verfügung steht:**
    - stimme voll und ganz zu (1)
    - stimme eher zu (2)
    - stimme eher nicht zu (4)
    - stimme überhaupt nicht zu (5

2. **Arme Länder sollten nicht nur den Zugang zum kostenlosen COVID-19-Impfstoff erhalten, sondern auch den kostenlosen Zugang zu anderen Impfstoffen erhalten.**
    - stimme voll und ganz zu (1)
    - stimme etwas zu (2)
    - stimme etwas nicht zu (4)
    - stimme überhaupt nicht zu (5)

Block 9: Wissen über COVID-19-Impfstoffentwicklung und Vertrauen in die Zulassungsbehörden (18 Fragen)

1. **Hat sich ein Impfstoffkandidat in Laborstudien bewährt, beginnen die Planungen für die klinischen Studien an freiwilligen gesunden Probanden (Testprozess). Wie lange sollte aus Ihrer Sicht ein solcher Testprozess mindestens dauern, damit Sie den Impfstoff für hinreichend sicher halten?**
    - 3-6 Monate (1)
    - 6 Monate bis zu einem Jahr (2)
    - 1-2 Jahre (3)
    - Zwischen 2 und 5 Jahren (4)
    - Mehr als 5 Jahre (5)

2. **Inwiefern stimmen Sie folgender Aussage zu: Studien zur Infektion des Menschen umfassen die absichtliche Infektion gesunder Freiwilliger. Solche Studien können besonders wertvoll für das Testen von Impfstoffen sein.**
    - stimme voll und ganz zu (1)
    - stimme etwas zu (2)
    - Teils/teils (3)
    - stimme etwas nicht zu (4)
    - stimme überhaupt nicht zu (5)

Derzeit befinden sich verschiedene Arten von Impfstoffen in der Entwicklung. Die folgenden Fragen beschreiben kurz die verschiedenen Impfstofftypen. Bitte bewerten Sie für sich ganz persönlich, wie Sie die verschiedenen Impfstofftypen als logischen, sicheren und wirksamen Weg zur Erlangung eines Schutzes (Immunität) gegen COVID-19 empfinden:

3. **Lebendimpfstoff (abgeschwächt): Diese Art von Impfstoff wird hergestellt, indem das Virus so geschwächt wird, dass es eine sehr kleine Infektion verursacht, genug, um Ihr Immunsystem zu aktivieren, aber sie werden dadurch nicht krank.**

- sehr positiv
- positiv
- weder noch
- negativ
- sehr negativ
- weiß nicht

4. **Tod- (inaktivierter) Impfstoff: Diese Art von Impfstoff wird hergestellt, indem das Virus so behandelt wird, dass es funktionsunfähig wird. Es infiziert Sie nicht, erfordert jedoch möglicherweise mehr Injektionen als ein Lebendimpfstoff.**

- sehr positiv
- positiv
- weder noch
- negativ
- sehr negativ
- weiß nicht

5. **Impfstoff gegen Untereinheiten: Diese Art von Impfstoff verwendet Virusstücke, die normalerweise in anderen Zellen wie Hefezellen hergestellt werden, um Ihr Immunsystem zu stimulieren. Es gibt keine Infektion, aber es kann mehr als eine Immunisierung erforderlich sein, um vollständig immun zu werden (3)**

- sehr positiv
- positiv
- weder noch
- negativ
- sehr negativ
- weiß nicht

6. **RNA-Impfstoff: Diese Art von Impfstoff verwendet genetische Moleküle des Virus, um Ihre Zellen anzuweisen, ein kleines Stück des Virus herzustellen. Ihr Immunsystem wird dann gegen dieses Virus aktiviert. Dies ist eine neuere Technologie, die jedoch wahrscheinlich eine starke Immunantwort hervorruft (4).**

- sehr positiv
- positiv
- weder noch
- negativ
- sehr negativ
- weiß nicht

7. **Trägerimpfstoff: Diese Art von Impfstoff verwendet ein anderes Virus, das beim Menschen keine Krankheit verursacht, um ein kleines Stück des COVID-Virus zu übertragen. Es wird eine sehr kleine Infektion verursachen; genug, um das Immunsystem zu aktivieren, aber dadurch werden sie nicht krank**
   - sehr positiv
   - positiv
   - weder noch
   - negativ
   - sehr negativ
   - weiß nicht

Table S1. Bitte beurteilen Sie bei den folgenden Aussagen, ob Sie damit übereinstimmen.

Fragen	Antwortformat	S
8. Ich mache mir Sorgen, dass ein COVID-19-Impfstoff bei mir eine COVID-19-Infektion verursacht.	Trifft voll und ganz zu → Trifft überhaupt nicht zu	0-5
9. Ich würde eine Immunität gegen COVID-19 lieber durch eine natürliche Ansteckung (z.B. bei einer infizierten Person) aufbauen, als mich impfen zu lassen.	Trifft voll und ganz zu → Trifft überhaupt nicht zu	0-5
10. Ich mache mir Sorgen um Nebenwirkungen des Impfstoffs.	Trifft voll und ganz zu → Trifft überhaupt nicht zu	0-5
11. Ich denke die Nebenwirkungen des Impfstoffs sind wahrscheinlich schlimmer als die COVID-19–Infektion selbst.	Trifft voll und ganz zu → Trifft überhaupt nicht zu	0-5
12. Ich würde mich eher impfen lassen, wenn ich dadurch wieder am sozialen und kulturellen Leben ohne Einschränkung teilnehmen könnte.	Trifft voll und ganz zu → Trifft überhaupt nicht zu	0-5
13. Ich würde mich impfen lassen wenn eine Impfung für internationale Reisen vorgeschrieben wäre.	Trifft voll und ganz zu → Trifft überhaupt nicht zu	
14. Ich würde mich eher impfen lassen, wenn ich weiß dass mein Umfeld sich auch gegen COVID-19 impfen lässt.	Trifft voll und ganz zu → Trifft überhaupt nicht zu	0-5
15. Ich würde mich eher impfen lassen wenn ich weiß, dass der COVID-19-Impfstoff in Europa oder in den USA entwickelt wurde.	Trifft voll und ganz zu → Trifft überhaupt nicht zu	0-5
16. Ich würde mich eher impfen lassen wenn ich weiß, dass der COVID-19-Impfstoff in Russland oder in China USA entwickelt wurde.	Trifft voll und ganz zu → Trifft überhaupt nicht zu	0-5
17. Ich mache mir Sorgen, dass ein COVID-19-Impstoff das Erbgut verändern kann.	Trifft voll und ganz zu →Trifft überhaupt nicht zu	0-5

18. Ich halte folgende Maßnahmen für wirksam, um mich und andere vor COVID-19-Infektion zu schützen (bitte alle zutreffenden Maßnahmen auswählen).

- Maske selber tragen (1)
- Andere Personen, die Masken tragen (2)
- Mindestabstand halten (Soziale Distanzierung (3)
- Häufiges Händewaschen (4)
- Desinfizieren von Oberflächen
- Corona-Warn-App auf Handys (5)
- Keine der oben genannten (6)

19. Ich ergreife derzeit regelmäßig die folgenden Maßnahmen, um mich und andere vor COVID-19 zu schützen (bitte alle zutreffenden Maßnahmen auswählen)

- Maske tragen in der Öffentlichkeit, wenn Mindestabstand nicht eingehalten werden kann (1)
- Regemäßige Einhaltung vom Mindestabstand (2)
- Öffentliche Hygienemaßnahmen wie häufiges Händewaschen und Desinfizieren von Oberflächen (3)
- Benutzung der Corona-Warn-App (4)
- Keine der oben genannten (5)

Block 10: Vertrauen (Regierung, Gesundheitseinrichtung).

1. Schränken Sie Ihre sozialen Kontakte wegen der Ausbreitung des Coronavirus ein?

- Ja, auf jeden Fall (1)
- Eher ja (2)
- Teils/teils (3)
- Eher nein (4)
- Nein, auf keinen Fall (5)

2. Wie zufrieden sind Sie mit der Qualität der Behandlung durch Ihren Hausarzt?

- Sehr zufrieden (1)
- Eher zufrieden (2)
- Eher unzufrieden (3)
- Sehr unzufrieden (4)
- den Hausarzt kenne ich noch nicht lang (5)

3. Wie problematisch ist COVID-19 in Deutschland?

- Überhaupt kein Problem (1)
- Im Vergleich zu anderen aktuellen Problemen unbedeutend (2)

- Ein ernstzunehmendes Problem (3)
- Ein schwerwiegendes Problem, wichtiger als die meisten anderen Probleme (4)
- Das wichtigste Problem, mit dem Deutschland derzeit konfrontiert ist (5)

4. Wie problematisch ist COVID-19 für Europa?

- Überhaupt kein Problem (1)
- Im Vergleich zu anderen aktuellen Problemen unbedeutend (2)
- Ein ernstzunehmendes Problem (3)
- Ein schwerwiegendes Problem, wichtiger als die meisten anderen Probleme (4)
- Das wichtigste Problem, mit dem Deutschland derzeit konfrontiert ist (5)

5. Wie problematisch ist COVID-19 für die Welt?

- Überhaupt kein Problem (1)
- Im Vergleich zu anderen aktuellen Problemen unbedeutend (2)
- Ein ernstzunehmendes Problem (3)
- Ein schwerwiegendes Problem, wichtiger als die meisten anderen Probleme (4)
- Das wichtigste Problem, mit dem Deutschland derzeit konfrontiert ist (5)

6. Wie zufrieden sind Sie mit der aktuellen Bewältigung der Pandemie durch die Bundesregierung?

- Sehr zufrieden (1)
- Eher zufrieden (2)
- Eher unzufrieden (3)
- Sehr unzufrieden (4)

7. Für wie angemessen halten Sie die von der Bundesregierung und den Landesregierungen in Deutschland ergriffenen Anti-Corona-Maßnahmen (Lockdown bzw. Teil-Lockdown mit strengen Kontaktbeschränkungen im Frühjahr 2020 und Winter 2020/21)? Bitte kreuzen Sie die Antwort an, die Ihrer Meinung am ehesten entspricht.

- Ich halte die Maßnahmen insgesamt für zu weitgehend und deshalb für unangemessen.
- Ich halte die Maßnahmen insgesamt für angemessen.
- Ich halte die Maßnahmen insgesamt für nicht weitgehend genug und deshalb für unangemessen.

8. Sollten Bundesregierung und Landesregierungen bei den Anti-Corona-Maßnahmen mehr oder weniger auf Empfehlungen und die Eigenverantwortung der Bürger setzen als auf staatliche, strafbewehrte Verbote?

- Bundesregierung und Landesregierungen sollten mehr als bisher auf die Eigenverantwortung der Bürger und auf Empfehlungen setzen statt auf strafbewehrte Verbote.

- Bundesregierung und Landesregierungen handeln bisher im Wesentlichen angemessen. Die Regierungen sollten weniger als bisher auf die Eigenverantwortung der Bürger und auf Empfehlungen setzen und mehr auf strafbewehrte Verbote.

9. Wie zufrieden sind Sie mit der aktuellen Arbeit des örtlichen oder regionalen Gesundheitsamtes bei der Pandemiebekämpfung?

- Sehr zufrieden (1)
- Eher zufrieden (2)
- Eher unzufrieden (3)
- Sehr unzufrieden (4)

10. Wie zufrieden sind Sie mit der aktuellen Arbeit der medizinischen Einrichtungen (Arztpraxen und Kliniken) bei der Pandemiebekämpfung?

- Sehr zufrieden (1)
- Eher zufrieden (2)
- Eher unzufrieden (3)
- Sehr unzufrieden (4)

11. Sollten sich weitere politische Maßnahmen eher auf den Gesundheitsschutz oder auf den Schutz der Wirtschaft richten?

- Eindeutig Schutz vor der Virus-Verbreitung (1)
- Eher Schutz vor der Virus-Verbreitung (2)
- Eher Wirtschaft am Laufen halten (3)
- Eindeutig Wirtschaft am Laufen halten (4)
- Weiß nicht (5)

12. Sind Sie mit der Art und Weise, wie die Demokratie in Deutschland funktioniert, alles in allem gesehen zufrieden?

- Sehr zufrieden
- Eher zufrieden
- Eher unzufrieden
- Sehr unzufrieden

Block 11: Soziodemographische Faktoren (9 Fragen)

1. Wann sind sie geboren

- Geburtsjahr________

2. In welchem Bundesland leben Sie?

- Baden-Württemberg
- Bayern
- Berlin
- Brandenburg
- Bremen
- Hamburg
- Hessen
- Mecklenburg-Vorpommern
- Niedersachsen
- Nordrhein-Westfalen
- Rheinland-Pfalz
- Saarland
- Sachsen
- Sachsen-Anhalt
- Schleswig-Holstein
- Thüringen

3. Geschlecht:

- Männlich (1)
- weiblich (2)
- Diverse (3)
- Keine Angaben (4)

4. Was ist Ihr Familienstand?

- Ledig (1)
- verheiratet (2)
- in einer festen Paarbeziehung(3)
- Verwitwet (4)
- Geschieden (4)
- Alleinerziehend (5)

5. **Wie würden Sie Ihren Gesundheitszustand im Allgemeinen beschreiben?**
   In den letzten 7 Tagen (heute eingerechnet) war er
   - sehr gut.
   - gut.
   - zufriedenstellend
   - weniger gut
   - schlecht.

6. **Wie viel Kinder unter 18 Jahre leben in ihrem Haushalt.**
   - 0 (1)
   - 1 (2)
   - 2 (3)
   - 3 (4)
   - Mehr als 3

7. **Was ist Ihr höchster erreichter Bildungsabschluss? (bei im Ausland erworbenen Abschlüssen bitte die vergleichbaren deutschen Abschlüsse ankreuzen)**
   - ohne Schulabschluss
   - Volksschulabschluss, Hauptschulabschluss
   - mittlere Reife
   - Fachabitur
   - Abitur
   - Fachhochschulabschluss
   - Hochschulabschluss
   - Promotion

8. **Welcher Religionsgemeinschaft gehören Sie an?**
   - Römisch-katholische Kirche
   - Evangelische Kirche, EKDKonfessionsgebundene Muslime
   - Christlich-Orthodoxe Kirchen
   - Sonstige christliche Gemeinschaften (z.B. Freikirchen, Neuapostolische Kirche, Zeugen Jehovas, Mennoniten)
   - Buddhismus
   - Judentum
   - Hinduismus
   - Jesiden
   - Sonstige Religionsgemeinschaften
   - Konfessionsfreie / ohne Religionszugehörigkeit

9. Welcher Partei stehen Sie traditionell am nächsten?

- CDU/CSU
- SPD
- GRÜNE
- FDP
- LINKE
- AfD
- Einer anderen Partei

10. Frage 66. Wie hoch ist das monatliche Nettoeinkommen Ihres Haushaltes insgesamt? Geben Sie bitte das Nettoeinkommen Ihres Haushalts vor der Corona-Pandemie an.

- unter 1250 €
- 1250 € bis unter 1750 €
- 1750 € bis unter 2250 €
- 2250 € bis unter 3000 €
- 3000 € bis unter 4000 €
- 4000 € bis unter 5000 €
- 5000 € und mehr

11. Gehören Sie einer oder mehrerer der folgenden „Corona-Risikogruppen" (laut Robert-Koch-Institut) an? Bitte wählen Sie alle Kategorien aus, die auf Sie zutreffen.

- Alter über 60 Jahre
- Übergewicht (BMI>30)
- Erkrankung des Herz-Kreislauf-Systems (z.B. koronare Herzerkrankung, Bluthochdruck, Herzinsuffizienz, Vorhofflimmern, Herzinfarkt, Schlaganfall)
- Chronische Lungenerkrankung (z.B. COPD, Lungenfibrose, Lungenemphysen, Sarkoidose, zystische Fibrose, Lungenfibrose, Schweres Asthma)
- Chronische Lebererkrankung
- Chronische Nierenerkrankung
- Diabetes mellitus Typ1 und Typ2
- Krebs
- Immunschwäche (z.B. durch Einnahme bestimmter Medikamente wie Glukokortikoide, Antikörperbehandlung, Lymphom, Leukämie, Amyloidose, Sichelzellanamie, angeborene Immundefekte, Stammzelltransplantation, Knochenmarktransplantation)
- Sonstige, nämlich--------

12. Üben Sie einen Beruf im Gesundheitssektor aus? (z.B. Krankenpfleger/in, Arzt/Ärztin, Apotheker/in, Labormitarbeiter/in, Altenpfleger/in, etc)

- Ja
- Nein

13. Falls Frage 65=Ja, welcher Berufsgruppe gehören Sie?

- Altenpfleger/in
- Apotheker/in
- Arzt/Ärztin
- Diätassistent/in
- Ergotherapeut/in
- Physiotherapeut/in
- Gesundheits- und Krankenpfleger/in
- Hebamme/Entbindungspflege
- Logopäde/-in
- MTA/CTA/BTA/PTA
- Rettungsfachpersonal
- Psychotherapeut/in

14. Wie zufrieden sind Sie gegenwärtig, alles in allem, mit Ihrem Leben?

- Sehr zufrieden (1)
- Eher zufrieden (2)
- Eher unzufrieden (3)
- Sehr unzufrieden (4)
